# Molecular Identification and Analysis of Human Enteroviruses Isolated from Healthy Children in Shenzhen, China from 2010 to 2011

**DOI:** 10.1371/journal.pone.0064889

**Published:** 2013-06-06

**Authors:** Wei Wu, Wen-Bo Xu, Long Chen, Hui-Ling Chen, Qu Liu, Dong-Li Wang, Ying-Jian Chen, Wei Yao, Gang Li, Bin Feng, Bai-Hua Shu, Yi-Kai Zhou, Ya-Qing He

**Affiliations:** 1 MOE Key Laboratory of Environment & Health, Institute of Environmental Medicine, School of Public Health, Tongji Medical College, Huazhong University of Science and Technology, Wuhan, Hubei, China; 2 Chinese Center for Disease Control and Prevention, Beijing, China; 3 School of Life Sciences, Shenzhen University, Shenzhen, China; 4 Shenzhen Center for Disease Control and Prevention, Major Infectious Disease Control Key Laboratory, Shenzhen, China; 5 Shenzhen Longgang District Center for Disease Control and Prevention, Shenzhen, China; 6 Shenzhen Guangming District Center for Disease Control and Prevention, Shenzhen, China; University of Hong Kong, Hong Kong

## Abstract

**Objective:**

To determine the prevalence and distribution of human enteroviruses (HEVs) among healthy children in Shenzhen, China.

**Method:**

Clinical specimens were obtained from 320 healthy children under 5 years old in Shenzhen, China from 2010 to 2011. The specimens were evaluated using real-time PCR and cell cultures. The positive specimens were further tested using reverse transcription-seminested PCR (RT-snPCR). Molecular typing and phylogenetic analysis were based on the sequence determined.

**Results:**

Among the 320 samples, 34 were tested positive for HEVs (10.6%) and 22 different serotypes were identified using RT-snPCR. PV1 and PV2 were also detected. The predominant serotype observed was EV71 (17.6%), followed by CV-B4 (14.7%). HEV-B was detected most frequently, with an overall prevalence of 47.1%. HEV-A and HEV-C were found in 32.3% and 20.6% of the samples, respectively. No HEV-D was identified. Molecular phylogeny indicated that all EV71 strains were of C4 genotype.

**Conclusion:**

Although a variety of HEVs was detected in healthy children, HEV-B was relatively more prevalent than other HEV species. Considering HEV-A is more prevalent than HEV-B among patients with hand-foot-mouth disease, additional long-term surveillance of HEV is warranted in both asymptomatic and symptomatic populations.

## Introduction

Enteroviruses are small, single-stranded, positive-sense RNA viruses from the genus *Enterovirus* of family Picornaviridae. Most enteroviruses are common causes of human infections [1], such as cutaneous and visceral infections, aseptic meningitis, and hand-foot-mouth disease, which are reportedly prevalent in many regions in China [2], [3]. Human enteroviruses were traditionally classified into poliovirus (PV), coxsackievirus (CV) A and B, echovirus (E), and numbered enteroviruses (EVs). Since 1970, the original classification for human enteroviruses has been modified based on the molecular and biological properties. This revised classification recognizes at least 100 subtypes, classified into four species: Human enterovirus A (HEV-A) species include EV71, CVA16, CVA2-8, CVA10, CVA12, CVA16 and EV89-92; HEV-B species include CVA9, CVB1-6, most echoviruses, and some new enteroviruses; HEV-C species consist of PV1-3, the majority of coxsackievirus A, and some new enteroviruses; HEV-D species only include EV68, EV70 and EV94 [4]. Enteroviruses cause illness in humans at any age and children <5 years old are the most susceptible.

The traditional method for detecting enteroviruses is based on conventional cell cultures using different cell lines to increase the sensitivity. However, isolating enteroviruses from stool is frequently unsuccessful because of the low viral titers in clinical specimens and the poor growth of some serotypes in cell cultures [5]. Thus, molecular diagnostic tests were used, which targets highly conserved sites in the 5′ untranslated region, allowing the detection of all members of the genus [6], and is more sensitive and much faster than traditional culture. To identify the serotype, a highly sensitive reverse RT-snPCR method was developed that amplifies all known human EV serotypes, permitting the detection of EVs in clinical specimens, and the identification of serotypes through sequence determination [7]. RT-snPCR shows good correlation with standard techniques, but it is rarely used routinely for identifying enteroviral serotypes in clinical diagnostic laboratories.

Although human enteroviruses (HEVs) are present in most countries, the largest disease outbreaks were seen in the Asia-Pacific region. Shenzhen, located on the southern coast of China, trades extensively with other regions in Southeast Asia and has a relatively high incidence of enteroviruses. Thus, to understand the epidemiology of enteroviruses, detecting enteroviruses among healthy children is of particular use. In the present work, we performed a two-year study to characterize and evaluate the prevalence of enteroviruses in healthy children under 5 years old. We systematically performed cell cultures, real-time PCR, and RT-snPCR to maximize enterovirus detection.

## Methods

### Stool Specimens

A total of 320 specimens were collected from healthy 3-year-old to 5-year-old children in kindergarten. Up to 16 stool specimens were randomly collected monthly from 2010 to 2011, except during summer vacation and Spring Festival. All specimens were stored at −80°C.

The study was approved by the ethics committee of the Shenzhen Center for Disease Control and Prevention, and was conducted in compliance with the principles of the Declaration of Helsinki. Written informed consents were obtained from the parents or legal guardians of the subjects.

### Processing of Stool Samples and Virus Isolation

The human epidermoid laryngeal carcinoma cells (HEP-2) and rhabdomyosarcoma cells (RD) were a gift from the Chinese Center for Disease Control and Prevention. A 10% stool extract, containing phosphate-buffered saline (PBS, pH 7.4), benzylpenicillin (100 U/mL), streptomycin sulfate (100 µg/mL), gentamicin sulfate (50 µg/mL), and chloroform, was prepared by shaking with glass beads. The resulting extract was centrifuged (1,200 ×g for 20 min), and the supernatant was collected and filtered (0.45 µm). Both the RD and the HEP-2 cell lines were cultured in Eagle’s minimal essential medium supplemented with 5% (v/v) and 2% (v/v) fetal bovine serum, respectively. The growth medium contained 20 mmol/L HEPES buffer (pH 7.4), 20 mmol/L MgCl_2_, benzylpenicillin (100 U/mL), streptomycin sulfate (100 µg/mL), gentamicin sulfate (50 µg/mL), and nystatin (50 U/mL). The cells were cultured in 24-well plates until they formed a monolayer. The culture (500 µL) was inoculated into 24-well plates at room temperature. The cultures were incubated at 37°C under a 5% CO_2_ atmosphere. The viral cultures were examined twice a week and considered positive if cytopathic effects (CPE) appeared within 21 days. Viral RNA was purified from 200 µL of virus-positive cell culture supernatant using a High Pure viral RNA kit (Roche, Germany) according to the manufacturer’s recommendations and used as the template for the subsequent real-time PCR.

### Extraction and Real-time PCR

Viruses obtained directly from the specimens were compared with those isolated from the cell cultures. Stool specimens were mixed thoroughly with 5 to 10 volumes of Hank’s balanced salt solution to yield homogeneous suspensions. The mixture was shaken vigorously for 20 min in a mechanical shaker, and centrifuged at 13,000 ×g for 5 min at room temperature to remove the solids. The resulting supernatant was transferred into a fresh tube. Viral RNA was extracted from the stool suspension supernatants using a High Pure viral RNA kit (Roche, Germany), according to the manufacturer’s recommendations. The extracts were eluted with 50 µL of nuclease-free water. The RNA extracted from the stool specimens and infected cell culture was immediately used as templates for real-time PCR, and the remaining samples were stored at −80°C until RT-snPCR amplification analysis. Real-time PCR was performed to detected EV71, CA16, and enterovirus universal (EVUN) (TaKaRa, Dalian, China) in all RNA samples. The assay was carried out in 25 µL reaction mixtures composed of 5 µL of cDNA and 20 µL of master mix. The master mix contained the primer pair and TaqMan probe. The one-step RT-PCR assay was performed on ABI 7500 system (Applied Biosystems, USA) in a 96-well format under the following conditions: reverse transcription at 50°C for 20 min, initial denaturation at 95°C for 5 min, followed by 40 cycles of amplification with denaturation at 95°C for 15 s, and annealing and extension at 60°C for 1 min. Real-time PCR was considered positive if the cycle threshold (ct) values were less than 40. The positive RNA from the viral cultures and specimens were subsequently subjected to RT-snPCR.

### RT-semi-nested PCR and Sequencing

Synthesis of cDNA was carried out in a 10 µL reaction mixture containing 5 µL of RNA, 100 µM each of the deoxynucleotide triphosphates (dNTP; TaKaRa, Japan), 0.01 M dithiothreitol, 1 pmol each of the cDNA primers (primer AN32, AN33, AN34, and AN35; Table 1), 20 U of RNasin (TaKaRa, Japan), and 100 U of Superscript II reverse transcriptase (TaKaRa). Following incubation at 22°C for 10 min, and 45°C for 50 min, the entire 10 µL RT reaction mixture was used in the first PCR (final volume, 50 µL) (PCR1), consisting of 25 µL of 2× GoTaq® Green Master Mix (Promega, USA), 50 pmol each of primers 224 and 222 (Table 1), 7 µL of RNase-free water, with 40 cycles of amplification (95°C for 30 s, 42°C for 30 s, 60°C for 45 s). One microliter of PCR1 products was added to a second PCR (PCR2) for seminested amplification. PCR2 contained 40 pmol each of primers AN89 and AN88 (Table 1), 25 µL of 2× GoTaq® HotStart Green Master Mix (Promega, USA), and 16 µL of RNase-free water, with a final volume of 50 µL. The HotStart Green Master Mix was activated by incubation at 95°C for 5 min, prior to 40 amplification cycles of 95°C for 30 s, 60°C for 20 s, and 72°C for 15 s. The reaction products were separated and visualized on 1.2% agarose gels, containing 0.5 µg of ethidium bromide per mL, and were purified from the gel using a QIAquick gel extraction kit (QIAGEN, Germany). Slight variations in the sizes of the PCR products (350 to 400 bp) were observed because of VP1 gene length differences in the different serotypes, as previously described [8], [9]. The resulting DNA templates were sequenced with a BigDye Terminator v1.1 ready reaction cycle sequencing kit on an ABI Prism 3100 automated sequencer (both from Applied Biosystems, USA), using the primers AN89 and AN88 or AN32 and AN33. The sequencing results were used in a BLAST search against the GenBank database.

**Table 1:** Primers used for cDNA synthesis, PCR amplification, and sequencing.

Primer	Sequence	Amino acid motif	Gene	Location
AN32	GTYTGCCA	WQT	VP1	3009–3002
AN33	GAYTGCCA	WQS	VP1	3009–3002
AN34	CCRTCRTA	YDG	VP1	3111–3104
AN35	RCTYTGCCA	WQS	VP1	3009–3002
224	GCIATGYTIGGIACICAYRT	AMLGTH(I/L/M)	VP3	1977–1996
222	CICCIGGIGGIAYRWACAT	M(F/Y)(I/V)	VP1	2969–2951
AN89	CCAGCACTGACAGCAGYNGARAYNGG	PALTA(A/V)	VP1	2602–2627
AN88	TACTGGACCACCTGGNGGNAYRWACAT	M(F/Y)(I/V)PPG	VP1	2977–2951

The size of the product was 350 bp to 400 bp.

### Phylogenetic Analysis

The sequences obtained in this study were included in a phylogenetic analysis, with reference strains of all enterovirus serotypes. Phylogenetic analysis was performed using the Kimura two-parameter model for nucleotide substitution and the neighbor joining method to reconstruct the phylogenetic tree using MEGA 5.0 [10]. The statistical significance of the phylogenies constructed was estimated by bootstrap analysis, with 1,000 pseudoreplicated data sets.

### Nucleotide Sequence Accession Numbers

Up to 34 new partial VP1 sequences were submitted to GenBank under accession numbers JX181899 to JX181932.

## Results

From 2010 to 2011, 320 healthy children [168 males (52.5%) and 152 females (47.5%)], with a mean (SD) age of 4.2 (0.6) years (range, 3–5 years) participated in the study. Of the 320, 37 (11.6%) showed CPEs in at least 1 cell line (Table 2). The viruses were isolated from cell cultures and then tested via EV71, CVA16, and Enterovirus universal (EVUN) Taqman real-time PCR. Finally, 6 (1.95%) viral strains tested positive under EV71 real-time PCR, 31 (9.5%) viral strains tested positive under EVUN real-time PCR, 4 viral strains tested positive under EV71 real-time PCR, but no viral strain tested under CVA16 real-time PCR. Thus, 33 (31+6–4) viral strains were confirmed positive using real-time PCR.

**Table 2:** Number of clinical specimens tested by Taqman real-time PCR and cell culture.

Real-time PCR	Cytopathic effect		Total
	Positive	Negative	
Positive	30	20	50 a
Negative	7	263	270
Total	37	283	320

a The qPCR results only include the viral RNA extracted from original specimens and exclude qPCR results of the viral RNA extracted from CPE-positive culture supernatants.

All viruses directly obtained from stool samples were also tested via EV71, CVA16, and EVUN real-time PCR assays. A total of 50 (15.6%) samples were confirmed positive using real-time PCR (Table 2). However, 30 of the cell culture samples tested positive. Therefore, only 53 samples (50+33–30), including those from cell cultures, tested positive for enteroviruses under real-time PCR.

**Figure 1:**
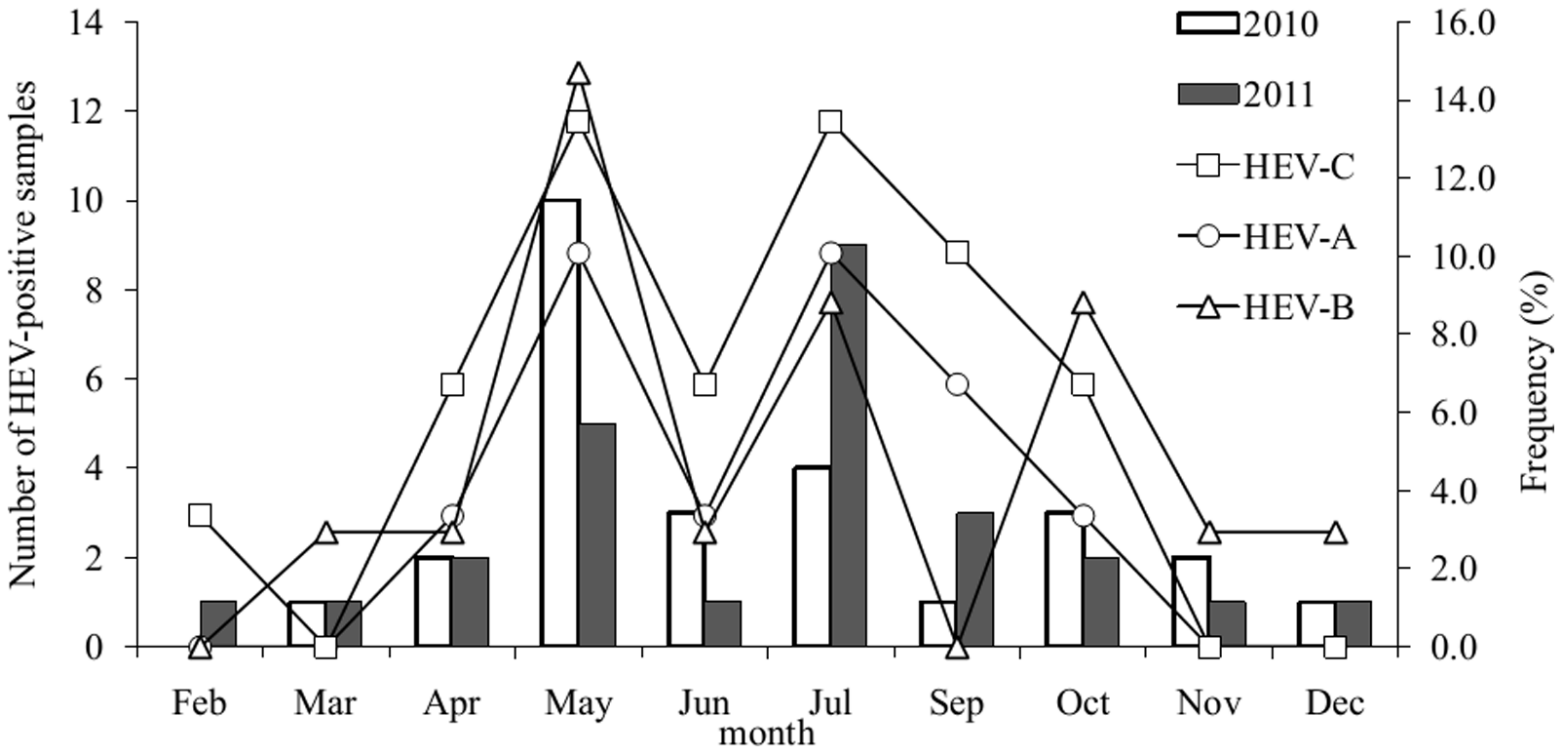
Monthly distribution of HEV-positive cases among healthy children in Shenzhen, China, 2010–2011. The bar represents the number of detected cases using real-time PCR, and lines represent the positive rates for HEV-A, HEV-B, and HEV-C during the two-year study. The curve was truncated in January because of the Spring Festival and in August because of the summer holiday.

The number of samples positive for enteroviruses confirmed via real-time PCR in the study is shown in Fig. 1. The average yearly seasonal incidence of enterovirus fluctuated considerably during the 2-year study, with a low incidence observed during winter and a high incidence from March to November, and peak incidence in summer and autumn. The monthly enterovirus positivity rate of the stool specimens ranged from 0.00% in February 2010 to 62.5% in 2010 and 56.3% in July 2011. The variations followed a typical pattern for enteroviral infections, with the highest rates in May and July of 2010 and 2011.

Of the 53 positive samples detected using real-time PCR, 64.2% (34/53) were assigned at the species level using RT-snPCR. The number of HEV findings, as well as the different species and serotypes are presented in Table 3. In 2010, 23 specimens were successfully sequenced, with CVB4 being the most prevalent serotype. In 2011, the number of EV sequences obtained was lower, and the most prevalent serotype was EV71. Among the specimens tested, HEV-A was detected in 11 samples, HEV-B in 16 samples, and HEV-C in 7 samples, accounting for 3.4%, 5.0% and 2.2% of the samples, respectively. No HEV-D viral groups were detected. Serotypes belonging to HEV-A, HEV-B and HEV-C species covered 6, 10, and 5 of the specimens, respectively. A total of 21 different HEV serotypes were identified. The most prevalent serotype was EV71, accounting for 17.6% of the HEV findings. Other prevalent serotypes were CVB4 (14.7%), CVA1 (8.8%), CVB2 (5.9%), CVB1 (5.9%). There were no significant differences were observed in the prevalence of HEV-A and HEV-B between boys and girls in the study.

**Table 3:** Number of HEV findings, different species, and serotypes isolated from 320 stool samples from 2010 to 2011 in Shenzhen District.

		2010(n = 21)	2011(n = 13)	Total(n = 34)	% Findings(100.0)	% Stool samples(10.6)
HEV-A	EV71	2	4	6	17.6	1.8
	CVA10	1	0	1	2.9	0.3
	CVA14	1	0	1	2.9	0.3
	CVA4	0	1	1	2.9	0.3
	CVA6	0	1	1	2.9	0.3
	CVA2	0	1	1	2.9	0.3
	Total	4	7	11	32.4	3.4
HEV-B	CVB4	5	0	5	14.7	1.5
	CVB2	2	0	2	5.9	0.6
	E6	1	0	1	2.9	0.3
	E14	1	0	1	2.9	0.3
	E13	1	0	1	2.9	0.3
	CVB5	1	0	1	2.9	0.3
	E25	1	0	1	2.9	0.3
	CVB1	0	2	2	5.9	0.6
	E12	0	1	1	2.9	0.3
	E1	0	1	1	2.9	0.3
	Total	12	4	16	47.1	5.0
HEV-C	CVA24	1	0	1	2.9	0.3
	CVA1	2	1	3	8.8	0.9
	PV1	0	1	1	2.9	0.3
	PV2	1	0	1	2.9	0.3
	EV96	1	0	1	2.9	0.3
	Total	5	2	7	20.6	2.2

**Figure 2:**
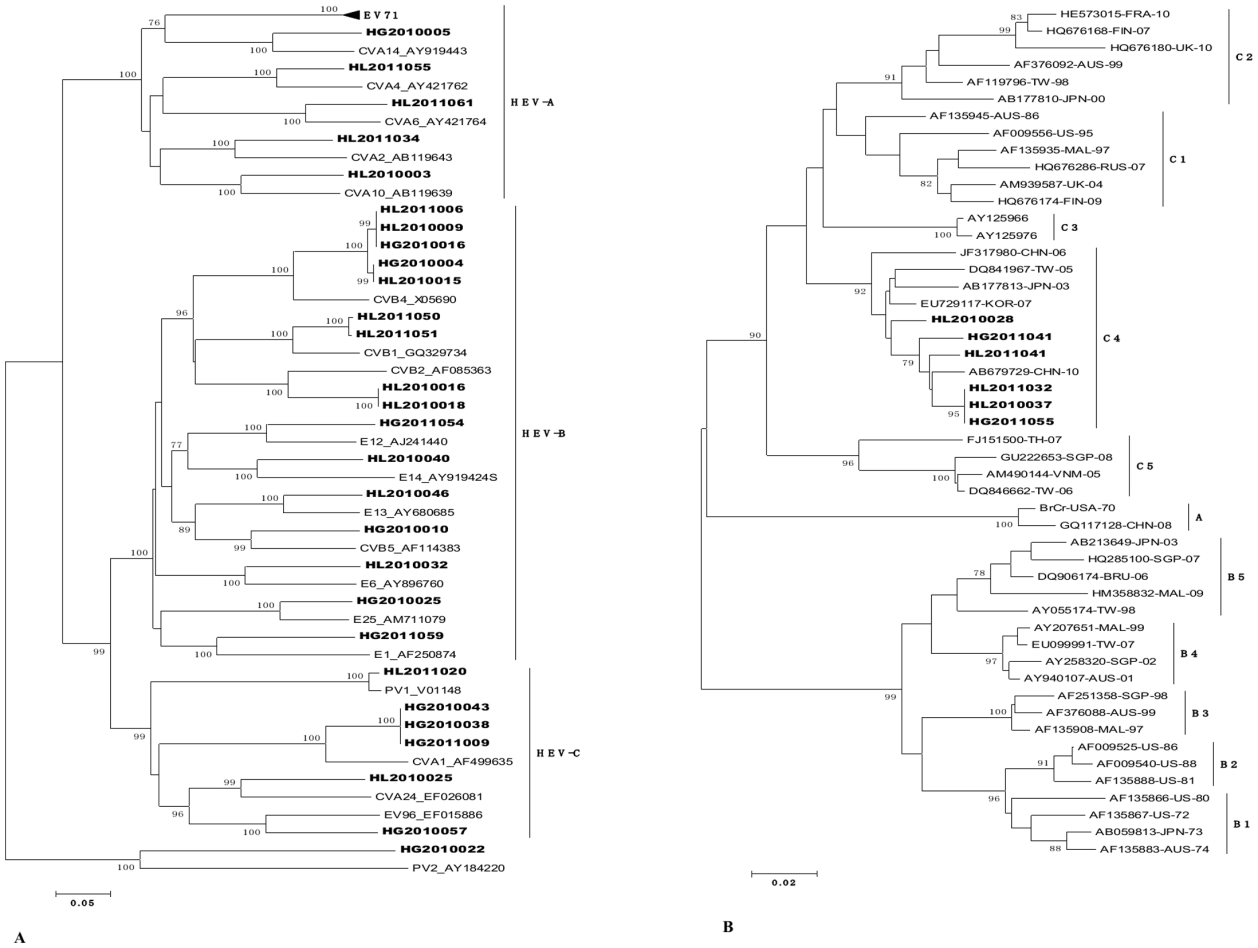
A. Rooted phylogenetic trees of partial VP1 sequences from clinical specimens collected from healthy children with EV infection from 2010 to 2011 and reference sequences available in GenBank for each serotype. The evolutionary distances were calculated using the Kimura two-parameter model for nucleotide substitution and the neighbor joining method to reconstruct the phylogenetic tree (MEGA version 5.0). Sequence names for field strains are constructed as follows: municipality number (starting with HL for the Longgang residential district of Shenzhen and with HG for the Guangming residential district of Shenzhen). The 21 reference sequences have GenBank accession numbers, whereas sequences generated in the present study are in bold letters. **B.** Phylogenetic tree depicting the relationships among the VP1 sequences of the EV71 isolates.

A phylogenetic tree was constructed from the aligned partial 5′ NC region sequences and homologous sequences from GenBank, to guide the choice of the set of primer pairs for the VP1 RT-PCR (tree available on request). All of the VP1 sequences obtained were used as query sequences for comparison with the sequences in GenBank. Each sample was assigned the serotype with the highest identity score. A phylogenetic tree was constructed based on the amino acid alignment of all VP1 fragments, including relevant reference strains from GenBank, using the neighbor joining method. In the VP1 tree (Fig. 2), clinical specimens of the same serotype clustered together with the respective GenBank reference sequences, supported by high bootstrap values, further confirming the serotype identification. All samples formed monophyletic clusters.

## Discussion

Enteroviruses (EV) are the most common viruses that infect humans. Rapid HEV detection and subsequent identification of the serotype in clinical cases are important for epidemiologic surveillance. Both traditional cell culture method and real-time PCR were used to maximize the number of positive samples. RT-snPCR was done using three primer pairs (Table 1), replacing primers 292 and 222, designed through the consensus degenerate hybrid oligonucleotide primer approach [11], [12]. Overall, 16.6% (53/320) of the stool samples tested positive using real-time PCR, and the serotypes were identified in 10.6% (34/320) of the total number of samples. This result is higher than in recent reports [13], [14], wherein only 4.8% of the specimens were HEV-positive in Swedish samples. In this study, 21 different enterovirus serotypes were detected, which suggests that enteroviral infections occur primarily in the Asia-Pacific region, and that the combination of cell lines, real-time PCR, and RT-snPCR is more efficient and accurate than traditional PCR that only uses primers 222 and 292.

The prevalence rates of HEV-A, HEV-B, and HEV-C were 3.4%, 5.0%, and 2.2%, respectively. HEV-D species were not detected in the samples. These findings are in accordance with other previous epidemiologic studies [15], [16]. The number of detected HEV-A is slightly lower than those in other studies [14], [17]. The most frequently identified HEV serotype was EV71, all of which was of the C4 subtype (Fig. 2 B). Since 1998, EV71 has been found in many cases and has recently become the predominant serotype in China, accounting for more than 100 deaths in 2009 [18]. It also caused 45 deaths in Hungary (1978) [19], and 29 deaths in Malaysia (1997) [20]. Other HEV-A species (CVA10, CVA14, CVA4, CVA6, and CVA2) were detected with only a single strain each. CVA10 and CVA6 are common etiologic agents of herpangina and they have been prevalent in Japan since 2005 [21], [22]. In Finland and Singapore, CVA6 and CVA10 are reportedly the predominant enteroviruses, causing a large outbreak of HFMD with onychomadesis [23], [24]. Surprisingly, no CVA16 strain was found in this study.

Human enterovirus B (HEV-B) is the most common and diversified enterovirus species, containing more than 30 serotypes of echoviruses (E), 6 serotypes of coxsackie B virus (CBV), coxsackievirus A9 (CVA9), and a number of newer enteroviruses. In this study, 10 strains of coxsackie B viruses and 6 strains of echoviruses were detected. The results for HEV-B are similar those by Witso [17], who reported an HEV-B prevalence of 4.8% in Norway, whereas Cook [25] reported a prevalence of 4.3% in Australia. However, considering other clinical manifestations, HEV-B species account for the highest percentage. In Taiwan, HEV-B species account for the highest percentage of studied cases (79.7%) [26]. In Yunnan, HEV-B species account for 75.2% [27]. CVB4 was the most frequent HEV-B serotype, comprising 14.7% of the findings. Several reports on newborn nursery outbreaks of non-polio enterovirus infection are present. Most outbreaks have been due to echovirus 11 or group B Coxsackie virus serotypes 1–5. In 1997, an outbreak in India was caused by Coxsackie B4, involving 20 neonates and 12 staff members, over an eight-month period [28]. In Korea (2008), echovirus type 30 (E30) and E6 have been associated with outbreaks and frequent meningitis [29]. Identification of HEV-B as the prominent EVs in Shenzhen indicates that much attention should be given to this genetic group.

Recent studies performed using sensitive RT-snPCR methods that directly used clinical samples as starting materials have shown a high prevalence of HEV-C infections, accounting for 20.6% of the findings. HEV-C species include several serotypes, namely: coxsackie A virus, PV1-3, and some new enteroviruses. The current study detected 7 HEV-C strains, namely, 1 CVA24, 3 CVA1, 1 PV1, 1 PV2, and 1 EV96. CVA24 is the causative agent of hemorrhagic conjunctivitis outbreaks [30]. Natural and engineered heterotypic recombinants of HEV-C have already been reported. Recombination between wild and vaccine strains has also been observed in type 1 wild vaccine recombinant PV, sharing a 367 nt block of a Sabin 1–derived sequence that spans the VP1 and 2A genes that circulated in China from 1991 to 1993 [31]. Several poliomyelitis outbreaks associated with vaccine-derived polioviruses (VDPVs) were reported in different parts of the world in recent years, particularly in Madagascar in 2002 [32]. These VDPVs appear to be recombinant viruses of vaccine polioviruses and HEV-C species. The first outbreak of poliomyelitis associated with VDPVs was reported in the Dominican Republic and Haiti from 2000 to 2001 [33]. Subsequent VDPV outbreaks occurred in the Philippines, China, Indonesia, Cambodia, Madagascar, and more recently in Myanmar and Nigeria, the Philippines, China, Indonesia, Cambodia, Madagascar [32], [34]–[36]. Most VDPVs are recombinants of PV and other HEV-C, primarily coxsackie A viruses [37], and the prevalence of coxsackie viruses in the current study is high. Improving viral surveillance and vaccination strategies is therefore important for preventing outbreaks of potential vaccine-derived recombinant polioviruses.

Enterovirus 96 (EV96) is a new HEV-C species, and its prototype strain was isolated from a stool specimen of an acute flaccid paralysis (AFP) patient, in 2000, in Bangladesh [38]. Furthermore, several other EV96 strains were reportedly isolated from AFP patients and healthy persons in Finland, Slovakia, and Yunnan Province in China [27], [39], [40], which implies that EV96 may have originated from East Asia or South Asia. Therefore, strengthening the surveillance of EV96 is essential in Shenzhen in southeast China.

The results also indicate clear seasonal variations in enterovirus infections in subtropical climates. Typically, the enterovirus season covers summer months and early autumn [30], [41]. According to the present study, enterovirus season extends from May to October. Among the HEV-positive specimens, 88.7% were collected during this period. Analysis of month-specific frequencies of HEV-A and HEV-B showed peaks in May and June (Fig. 1). These findings are consistent with previous reports [14] that both HEV-A and HEV-B have highest prevalence during the aforementioned period.

We also speculate that the differences in the prevalence of individual HEV serotypes between our study and others reflect methodological differences. To our knowledge, few studies have reported that the prevalence of HEV-B is higher than HEV-A in clinical samples using molecular methods. PV1 and PV3 are even rarely detected in healthy children. However, the prevalence of HEV-B and HEV-C in this study reached 47.1% and 20.6%, respectively. Thus, RT-snPCR methods are more sensitive for HEV-B and HEV-C.

In this study, we present a detailed report of the detection of enteroviruses in healthy children under 5 years old in China. The results suggest that several enterovirus serotypes are relatively common in our region, particularly during summer months, among healthy children. Certain serotypes, particularly EV71 and CVB4, require more rigorous surveillance in terms of the potential for treatment and prognostication.
